# Complete Anatomy

**DOI:** 10.5195/jmla.2019.853

**Published:** 2020-01-01

**Authors:** Sara K. Motsinger

**Affiliations:** Medical Librarian, D’Angelo Library, Kansas City University of Medicine and Biosciences, Kansas City, MO, smotsinger@kcumb.edu

## PURPOSE

Complete Anatomy is an educational three-dimensional (3D) anatomy platform created by 3D4Medical, which has been developing medical products since 2009. Their content is vetted by an academic review board consisting of anatomical experts and clinical practitioners who practice in hospitals and teach in academic institutions worldwide. It is an anatomy platform used by students, educators, medical professionals, and institutions and has more than one million users of its interactive anatomy models, clinical video animations, and virtual dissection tools. In the spring of 2019, Complete Anatomy became a publishing platform for interactive anatomy courses [1]. These can be used by academic faculty when they are designing a curriculum, but also by students as stand-alone courses to review material. The Complete Anatomy app, winner of an Apple Design Award, is a dynamic way for students to explore anatomy beyond the traditional atlas.

## CONTENT AND USABILITY

**Figure 1:**
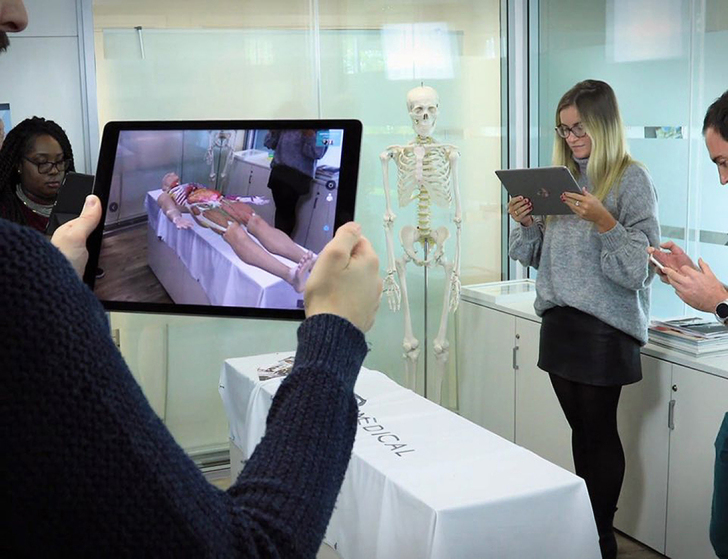
Visualization of Complete Anatomy augmented reality mode Image courtesy of Complete Anatomy.

Content varies by subscription, but all subscriptions center around the core interactive anatomy atlas and its features, including gross anatomy models, microscopic models of tissues and cells, a beating heart, realistic muscle motion, and bone mapping, with clear labeling of parts, surfaces, and landmarks. Users can isolate body regions, trace the pathways for arteries and nerves, and view nerves with the Innervation Pathway tool. With just a few taps, this tool can help users view the innervating nerves of a muscle and isolate or hide the path to visualize that muscle’s nerve supply. Users can further customize their view of the body by selecting one or more of twelve body systems, including skeletal and connective tissues, muscular, vascular (venous and arterial), lymphatic, nervous, respiratory, digestive, endocrine, urogenital, and integumentary. The anatomical model can appear to be placed virtually onto any flat surface using Augmented Reality mode. A multiuser Augmented Reality mode is also available (Figure 1). A full female model is currently under development; however, Complete Anatomy does offer a female thorax and pelvis.

Medical educators can use preset dissections in lectures and online materials as well as more than 1,500 videos and audio recordings, including videos grouped together for cardiology, orthopedics, ophthalmology, dentistry, and fitness. Screens are interactive lecture slides that can be used as is or further personalized for classroom use. Educators also benefit from the Curriculum Manager module, available with the purchase of the Educator license, that unlocks access to interactive learning resources as well as the ability to create learning resources that can then be published and shared with other educators. The Curriculum Manager acts like a learning management system, enabling educators to create specific groups to share courses with. A Dashboard feature is included that highlights student activity such as quiz results, attendance, and progress through the shared materials.

**Figure 2:**
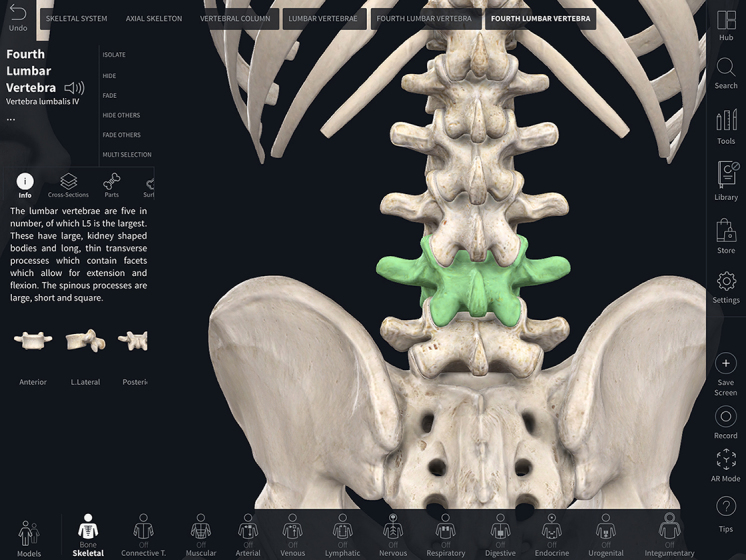
Detail of the fourth lumbar vertebra in skeletal view on the Complete Anatomy App Image courtesy of Complete Anatomy.

Navigating the Complete Anatomy app on a mobile device is an intuitive process, made easier by first utilizing the tutorials that are available on the 3D4Medical website. The selected anatomic model is easily manipulated and rotated with fingers on a mobile device or a mouse and keyboard on a computer (Figure 2). Selecting an anatomical structure provides the user with information about it, an audio recording to hear correct pronunciation, and tools for navigating that structure. Selecting the Motion option on a structure will enable the motion mode that shows realistic structural motion and articulation. The beating heart can be turned on or off with a double tap of the fingers or double click of the mouse.

The Dashboard, or main menu, is referred to as the “Hub” and can be accessed easily from the model view. Also useful is the “Recents” bar, which slides in or out of the left-hand side of the app while users are viewing the “Hub,” making it easy to locate items users viewed during previous sessions. Tips can be accessed by clicking the question mark in the model view and can help users navigate the multitudes of functionality options with brief video walkthroughs. Videos and specific content in the Complete Anatomy Library can be favorited by tapping a heart icon, although in this reviewer’s opinion, locating these items again is not intuitive and requires some digging.

When navigating the gross anatomy models, a variety of tools can be utilized: annotation tools, cutting tools (for both stripping away layers and adding fractures), and growth tools (including bone spurs), as well as a pain sphere to help visualize pain spots on specific areas of the body. Specific anatomic areas can be easily searched using the search function from any screen. Models are available offline; files will be synced the next time the user is connected to the Internet. Courses, Content, and Videos require Internet access.

The Systems menu can be accessed along the bottom of the screen. Utilizing the plus and minus buttons above each system allows navigation through that system’s layers. Clicking or tapping on the system buttons can quickly turn a system on or off.

## COURSES

While educators can create course content using the Curriculum Manager, they also have access to completely built courses in the Complete Anatomy Library. Courses are a part of the premium subscription levels (Student Plus, Educator, or Professional licenses). Current offers include 15 courses that feature more than 150 hours of lecture and video that can be used as supplemental material or assist with exam review. Course content will be doubling within the year.

Educators and students can make use of 130 pre-built quizzes, color-coded by difficulty in the Complete Anatomy Library, with user ratings displayed prominently in the thumbnail view. Individual quiz questions can also be viewed, added to Groups, and edited in those groups.

## INTENDED AUDIENCE

The app can be used by students as they learn, by educators as they build lectures and deliver relevant material, by medical professionals as they educate their patients, and by institutions that wish to offer Complete Anatomy as a resource for curriculum, education, and more. How the Curriculum Manager can be integrated into existing university learning management systems remains unclear to this reviewer at this time, though rollout of some Canvas (a popular educational learning management platform) functionality will be forthcoming. In this reviewer’s opinion, at this time, Complete Anatomy is an excellent learning tool with some teaching functionality. It appears that current and future developments are aiming toward Complete Anatomy becoming an educational tool with a more robust platform for those who wish to use it to teach.

## TECHNICAL REQUIREMENTS

Complete Anatomy is available for iPad (Air, Pro, 2017 or newer basic iPad, iPad mini 4, iPad OS 10.3, or later); Mac (MacBook late-2009 or newer, MacBook Pro mid-2010 or newer, MacBook Air late-2010 or newer, Mac mini mid-2010 or newer, iMac late-2009 or newer, and Mac Pro mid-2010 or newer, operating system 10.12 or later); Windows (operating system Windows 10); iPhone (iPhone 5s to newest model, operating system 10.3 or later); and Android phones (Android 7.0 or later) [2]. Three-day free trials are available so that users can test performance of the product on their devices before purchasing.

## COMPARISON TO SIMILAR PRODUCTS

Medical students use anatomy apps to help them understand anatomical structures because they are a convenient and modern tool that can supplement traditional educational delivery methods. In this reviewer’s opinion, Complete Anatomy has a wider depth and breadth of offerings than other anatomy platforms that are currently available (e.g., Primal Pictures, Visible Body) as well as more detail in its models. Other anatomy apps do not offer the ability to share content and have fewer tools with which to dissect and label models. While the pricing is higher than other similar offerings, it is a solid investment for those wishing to utilize a beautifully designed tool for bringing anatomy to life.

## CONCLUSION

Complete Anatomy is a robust and impressive resource for students who wish to supplement their traditional medical education with 3D models and tools to reinforce their learning in the classroom and lab. Using Complete Anatomy is a way to stay in touch with learning in the anatomy lab by making the cadaver portable on mobile devices. For educators, Complete Anatomy can lay the groundwork for building curricula with pre-built lessons and slides from anatomy educators across the globe. For educators operating in a learning management platform (e.g., Canvas or Blackboard), it is yet to be seen how the educational functionality will work with existing systems.

## 

**Sara K. Motsinger,** smotsinger@kcumb.edu, Medical Librarian, D’Angelo Library, Kansas City University of Medicine and Biosciences, Kansas City, MO
